# Nanomaterial Databases: Data Sources for Promoting Design and Risk Assessment of Nanomaterials

**DOI:** 10.3390/nano11061599

**Published:** 2021-06-18

**Authors:** Zuowei Ji, Wenjing Guo, Sugunadevi Sakkiah, Jie Liu, Tucker A. Patterson, Huixiao Hong

**Affiliations:** National Center for Toxicological Research, U.S. Food and Drug Administration, Jefferson, AR 72079, USA; Zuowei.Ji@fda.hhs.gov (Z.J.); Wenjing.Guo@fda.hhs.gov (W.G.); Suguna.Sakkiah@fda.hhs.gov (S.S.); Jie.Liu1@fda.hhs.gov (J.L.); Tucker.Patterson@fda.hhs.gov (T.A.P.)

**Keywords:** nanomaterial, database, physicochemical property, bioactivity, characterization

## Abstract

Nanomaterials have drawn increasing attention due to their tunable and enhanced physicochemical and biological performance compared to their conventional bulk materials. Owing to the rapid expansion of the nano-industry, large amounts of data regarding the synthesis, physicochemical properties, and bioactivities of nanomaterials have been generated. These data are a great asset to the scientific community. However, the data are on diverse aspects of nanomaterials and in different sources and formats. To help utilize these data, various databases on specific information of nanomaterials such as physicochemical characterization, biomedicine, and nano-safety have been developed and made available online. Understanding the structure, function, and available data in these databases is needed for scientists to select appropriate databases and retrieve specific information for research on nanomaterials. However, to our knowledge, there is no study to systematically compare these databases to facilitate their utilization in the field of nanomaterials. Therefore, we reviewed and compared eight widely used databases of nanomaterials, aiming to provide the nanoscience community with valuable information about the specific content and function of these databases. We also discuss the pros and cons of these databases, thus enabling more efficient and convenient utilization.

## 1. Introduction

With the rapid development of nanotechnology, various but similar definitions of nanomaterials have been proposed [1,2,3]. From the current available definitions of nanomaterials, summarized by Kreyling et al., most of them define nanomaterials based on the size parameter [4]. In this article, we used the definition from the EC Scientific Committee on Emerging and Newly Identified Health Risks. A manufactured nanomaterial is a material which is intentionally generated such that it is composed of discrete structural and functional parts, either at the surface or internally, with one or more dimensions at the order of 100 nanometers (nm) or less [5], exhibiting distinct and superior physicochemical and biological properties compared to their conventional equivalents [6]. The improved nanoscale properties such as hardness, electrical conductivity, magnetic characteristics, chemical reactivity, and toxicity are derived from a number of parameters such as shape, surface chemistry, size, and specific surface area [4,7,8,9]. So far, engineered nanomaterials have been proposed for a wide array of industrial applications such as paints, coatings, electronics, energy, power, cosmetics, and pharmaceuticals [10,11,12,13,14,15,16,17,18,19,20,21,22]. According to a recent report published by Grand View Research, Inc., the size of the global nanomaterials market is estimated to reach USD 22.9 billion by 2027 with a compound annual growth rate of 13.1% [23].

**Figure 1:**
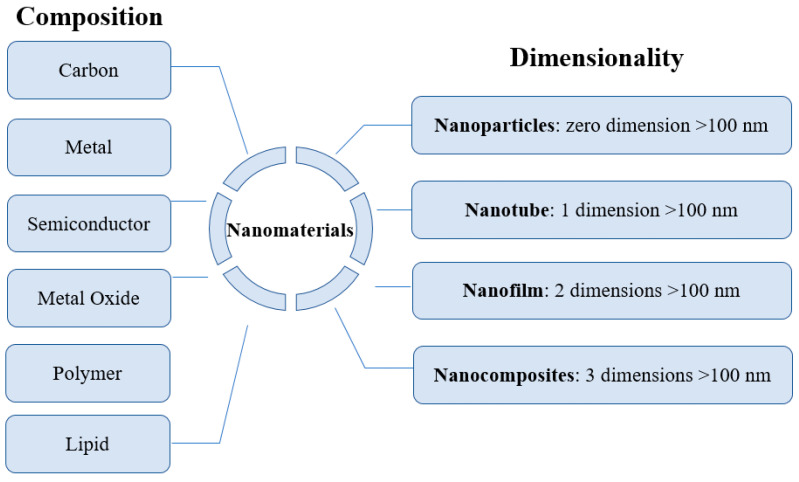
Classification of nanomaterials based on composition and dimensionality.

The rapid development of nanotechnology generates numerous nanomaterials with different properties and functions. To promote better development of nanotechnology, some basic and common terms and concepts have been proposed. For example, regarding the synthesis of nanomaterials, there are two basic approaches to synthesize materials with nanoscale features and attributes [24]. One is known as top-down fabrication, where small features are created based on large substrates using methods such as lithography, chemical ablation, laser ablation, and electrochemical carbonization [25,26,27,28]. Another one is called bottom-up fabrication, which assembles small building-block units into larger nanostructures. The bottom-up route includes self-assembly, microwave irradiation, hydrothermal/solvothermal treatment, and so on [29,30,31]. The synthesized nanomaterials, depending on their composition, can be divided into carbon-based nanomaterials, metal nanomaterials, semiconductor nanomaterials, metal oxide nanomaterials, polymer nanomaterials, lipid-based nanomaterials, and others, as shown in Figure 1. Nanomaterials can also be classified by their dimensionalities, including zero dimensional (0D: zero dimension > 100 nm) nanomaterials such as nanoparticles, one-dimensional (1D: one dimension > 100 nm) nanomaterials such as nanotubes, two-dimensional (2D: two dimensions > 100 nm) nanomaterials like graphene, and three-dimensional (3D: all three dimensions > 100 nm) nanomaterials, e.g., nanocomposites [32].

Considering both the mass production of nanomaterials and public health, numerous studies regarding their physicochemical properties, toxic effects, and environmental risks have been performed [33,34,35,36,37,38,39,40,41,42,43]. To give a few examples, the study performed by Magrez et al. showed that the cytotoxic effects of carbon-based nanomaterials (carbon nanofibers, carbon nanoparticles, and carbon nanotubes) were size-dependent, and the hazardous effects were enhanced if the functionalization of these nanomaterials was via acid treatment [44]. In another study, Fairbairn et al. studied the effects of metal oxide nanomaterials on sea urchin development. Their results suggested that sea urchin embryos are severely affected by ZnO nanomaterial treatment, while they are not sensitive to CeO_2_ or TiO_2_ nanomaterials under the tested conditions [45]. From these studies on nanomaterials, data have been generated, which makes analyzing and designing nanomaterials possible and easier. Thus, how to properly and effectively utilize these data become unavoidable questions in the nanoscience community. 

To take full advantage of these valuable resources, databases that can store and manage the data in a more organized way have been developed to help scientists study and design nanomaterials to meet their specific needs. Various nanomaterial databases are available online. However, there are no guidelines for scientists to select the appropriate databases when performing a specific area of research. Therefore, understanding and comparing the content, structure, function, advantage, and limitation of these databases become important and necessary for better utilization of them in nanoscience research. 

The primary objective of this review is to provide information for selection of the appropriate databases when conducting certain aspects of nano-research, enabling more convenient and efficient extraction of the nanomaterials-related data. To achieve this objective, eight popular nanomaterial databases including PubVINAS, caNanoLab (cancer Nanotechnology Laboratory), eNanoMapper, NR (Nanomaterial Registry), NBIK (Nanomaterial-Biological Interactions Knowledgebase), NKB (NanoCommons Knowledge Base), NIL (Nanoparticle information library), and Nanowerk were reviewed and systematically compared. According to our comparisons, the NR, eNanoMapper, and PubVINAS databases contain large amounts of data on physicochemical properties of nanomaterials, the caNanoLab and eNanoMapper databases provide biological experiments and relevant protocols, and the caNanoLab database also includes detailed descriptions of experimental designs.

## 2. Brief Description of the Databases

Many nanomaterial databases have been developed. After exploring their accessibility and data abundancy, eight databases (caNanoLab, eNanoMapper, NR, NBIK, NKB, NIL, Nanowerk, and PubVINAS) were found to be publicly accessible and contained rich information on various aspects of nanomaterials. These databases should be informative to the scientists in the community of nanoscience. Therefore, to help scientists better utilize them, we briefly describe the aspects of objective, data abundancy, and function of these databases. The basic information including websites, nanomaterials recorded, and major features of the eight databases is provided in Table 1.

The caNanoLab is a nanomaterial database that facilitates nanotechnology development in biomedicine by enabling information sharing across the international biomedical nanotechnology community [46,47]. The database has 1383 unique nanomaterial data records. Users can narrow down the data records by specifying nanomaterial entity, functionalizing entity, characterization type, and function of the nanomaterial of interest. The caNanoLab contains detailed information about the experimental design, composition, characterizations (physicochemical, in vitro, in vivo, and ex vivo) and publications of nanomaterials. The physicochemical properties of nanomaterials include size, shape, composition, purity, molecular weight, surface area, and relaxivity. Biological experimental data such as cytotoxicity, genotoxicity, oxidative stress, immunotoxicity, and pharmacokinetics are collected in this database. The experimental data can be exported in JSON, XML, and XLSX formats. Furthermore, caNanoLab supports the annotation of nanomaterials with characterizations and guarantees the sharing of the data in a secure manner.

eNanoMapper, supporting the collaborative safety assessments for engineered nanomaterials, was developed in the eNanoMapper project funded through the European Seventh Framework Programme [48,49]. It creates an infrastructure not only for data sharing and data analysis, but also for building computational toxicology models for engineered nanomaterials [50]. It is noteworthy that eNanoMapper integrates data from several data sources such as caNanoLab. In eNanoMapper, physicochemical properties such as size distribution, surface area, stability, freezing/melting point, zeta potential, shape, and aspect ratio are included. Furthermore, the availability and completeness of some nanomaterials and their physicochemical properties determined by experiments have been assessed [51]. eNanoMapper also contains a variety of toxicological experimental data such as cell viability, oxidative stress, immunotoxicity, genetic toxicity, and omics data. The detailed experimental protocols that were used to generate the toxicological data can be retrieved via the references included in this database. Various database functionalities have been implemented in eNanoMapper, including search, ontology annotation, data import and export through a web browser interface, and a REpresentational State Transfer (REST) web services application programming interface (API) (http://enanomapper.github.io/API/, accessed on 16 February 2021), facilitating the building of user-friendly features. Data in eNanoMapper can be exported in JSON, CSV, XML, JSON-LD, and XLSX formats. 

NR is a public and fully curated database that is funded by the National Institutes of Health (NIH) [52]. It archives experimental data such as biological and environmental effects of nanomaterials. The data are curated from multiple sources including caNanoLab, NBIK, and NIL. This database provides links to the original data sources. In this database, the nanomaterials can be browsed by their material type (e.g., metal, metal oxide, carbon, polymer), size (e.g., <25 nm, 25–74 nm, 75–149 nm, 150–300 nm, and >300 nm), shape (1D, 2D, and 3D), or surface area (e.g., <10 m^2^/g, 10–49 m^2^/g). NR contains a variety of physiochemical characterizations such as size, size distribution, aggregation, surface area, shape, composition, purity, surface charge, surface chemistry, surface reactivity, solubility, and stability. In addition, 608 biological studies (82% in vitro and 18% in vivo) are recorded in this database. The data can be downloaded in an easy-to-analyze Excel spreadsheet format. This database supports search, browse, comparison, and data retrieval of nanomaterials. 

Nanowerk is an online portal that provides rich information on nanoscience and nanotechnologies. The nanomaterial database in Nanowerk contains commercially available nanomaterial products and information on their vendors worldwide. This database comprises hundreds of suppliers of 3872 unique nanomaterials, including fullerene, graphene, nanofibers, nanoparticles (e.g., binary compound nanoparticles, complex compound nanoparticles, and single element nanoparticles), nanotubes (carbon nanotubes and non-carbon nanotubes), nanowires, and quantum dots. The data recorded in this database include component, size, and phase of the manufactured nanomaterials. Moreover, users can request a quote or contact the suppliers directly using the provided links after finding the nanomaterials of interest.

NBIK is a knowledgebase established by Oregon State University for understanding nanomaterial exposure risks by exploring the relationship between the physicochemical properties of nanomaterials and the biological interactions caused by exposure to nanomaterials. NBIK has 147 unique nanomaterials covering seven material types, including carbon, cellulose, dendrimer, metal, metal oxide, polymer, and semiconductor. The nanomaterials can be searched using material type, core (e.g., copper, gold, carbon), surface chemistry (shell composition and functional groups), shape (e.g., conical, cubic, dendritic), size range, and charge (e.g., +, − and 0). In NBIK, the biocompatibility data of the nanomaterials are obtained from testing with zebrafish embryos as the metric. The zebrafish embryo testing data for all the nanomaterials are presented in a heatmap. Similar to NR, NBIK also supports data export in the XLSX format.

NIL is a web-based nanoparticle information library. It was developed by the National Institute for Occupational Safety and Health (NIOSH) [53]. NIL provides a tool for sharing and searching health and safety-associated properties of nanoparticles. It contains information on composition, method of production, particle size, surface area, morphology (include scanning, transmission, and other electron micrographic images), availability for research or commercial applications, and associated or relevant publications of nanoparticles. This database can be browsed and searched with a set of functions, including origin search, structure search, element search, and size search. Currently, it only has 88 unique nanomaterials. 

NKB provides an openly accessible and sustainable nano-informatics framework for the assessments of the risks of nanomaterials. It was developed by Biomax Informatics AG, a bioinformatics software company. This knowledge base contains physicochemical properties such as size, size distribution, shape, coating, dynamic light scattering, polydispersity index, zeta potential, electrophoretic mobility, energy band gap, and geometric surface area of 598 unique nanomaterials. Two types of toxicity data, no observed adverse effect level and toxicity, are included in NKB. The data can be exported in Excel or a tab delimited text file. This database has search, analysis (e.g., RNA-Seq analysis, corona analysis, and image analysis), ontology browsing, data export, and data upload functions.

The data curated in the above-mentioned seven nanomaterial databases are not ideal for in silico modeling. For instance, some nanomaterial entities in the databases, such as structure, physicochemical properties, and biological endpoints, exist in text outputs without nanostructure annotations, which limited the application of supervised in silico modeling in predicting structure and toxicity correlation of nanomaterials. PubVINAS was developed to overcome the challenges in facilitating modeling of nanomaterials by providing the annotated nanostructures [54]. This database contains 12 material types (gold nanoparticles, silver nanoparticles, platinum nanoparticles, palladium nanoparticles, metal oxide nanoparticles, quantum dot nanoparticles, carbon nanotubes, peptide nanotubes, dendrimers, DNA origami, C60, and carbon nanoparticles), 725 unique nanomaterials, and 2142 nanodescriptors. The data in PubVINAS, including the physicochemical properties (e.g., size, shape, ligands number, logP, and zeta potential) and biological activities (e.g., cytotoxicity, cell uptake, cell viability, cell association, nonspecific/specific binding with AChE enzyme, protein adsorption, and oxidative stress) of nanomaterials, are extracted from thousands of scientific papers. These data were annotated and stored in the protein data bank (PDB) format files, which could be accessed from their web portal. Experimental protocols associated with the data are included in the database. Some machine learning models for predicting the properties (e.g., zeta potential, logP, and cellular uptake) of nanomaterials were also established based on those descriptors.

## 3. Comparative Analysis of the Databases

Although Nanowerk has the largest quantity of nanomaterials, considering that it mainly provides information about the vendors of commercialized nanomaterials, and it does not have biological characterizations of the nanomaterials, we excluded it from our comparative analysis. Meanwhile, it is noteworthy that some databases share data with each other. For instance, some data in the NR database are from caNanoLab, NBI, and NIL. Similarly, the eNanoMapper database also has data from caNanoLab.

In terms of the quantity of total nanomaterials, it was observed that the eNanoMapper, NR, and caNanoLab databases have more nanomaterials than the others. Each database has its own method of categorizing nanomaterials. Some databases just simply list each individual nanomaterial instead of grouping the nanomaterials. To make the comparative analysis of the databases clearer to researchers, we grouped the nanomaterials in each database into six categories based on their chemical composition: carbon-based nanomaterials, lipid-based nanomaterials, metal nanomaterials, metal oxide nanomaterials, polymeric nanomaterials, and semiconductor nanomaterials. The numbers of nanomaterials for the six categories in the seven databases were counted, and the results are listed in Table 2. The nanomaterials that could not be put into the six categories are listed as “Other” in Table 2. The comparative analysis revealed that caNanoLab, eNanoMapper, and NR not only contain large numbers of nanomaterials, but also cover all nanomaterial categories defined in this paper. The other four smaller databases have no lipid nanomaterials and fewer polymer nanomaterials.

**Figure 2:**
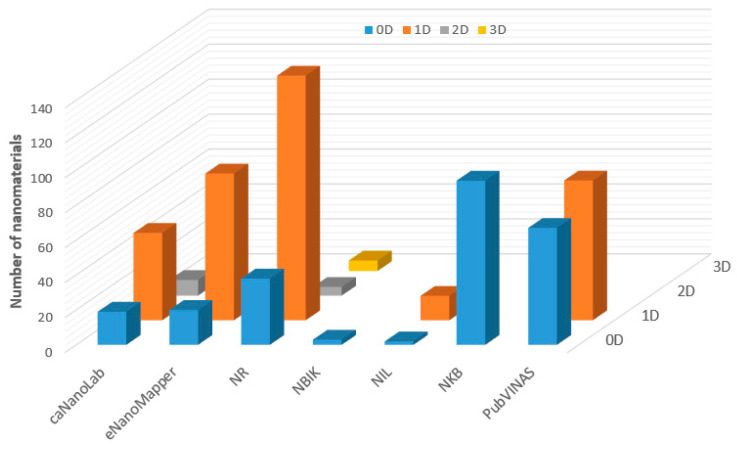
Number of carbon-based nanomaterials (*z*-axis) of four shape types (depicted in different colors and marked at the *y*-axis) in the seven nanomaterial databases indicated on the *x*-axis.

In addition, structures of the nanomaterials with the same composition can be further categorized by their dimensionalities (nanomaterials without dimension information are not included) as illustrated in Figure 1. Most of the nanomaterials contained in these databases are nanoparticles and nanotubes. Among the nanomaterials with the six compositions shown in Table 2, lipid-based nanomaterials are included only in NR, caNanoLab, and eNanoMapper. All lipid-based nanomaterials collected in these databases are nanoparticles. All seven databases have semiconductor nanomaterials. All semiconductor nanomaterials in these databases are nanoparticles, except for NR, which has 21 nanotubes and more than 200 nanoparticles. NIL does not have metal oxide nanomaterials. All metal oxide-based nanomaterials collected in the other six databases are nanoparticles, except for NR, which has 26 nanotubes and more than 400 nanoparticles. No polymeric nanomaterials are included in NIL and NKB. Most of the polymeric nanomaterials contained in the four other databases are nanoparticles; only PubVINAS and NR have a few nanotubes of polymeric nanomaterials. The majority of the metal nanomaterials are nanoparticles and are included in all seven databases. NR, caNanoLab, NIL, and NBIK also have some metal-based nanotubes. Only NIL has a few nanofilms. The carbon-based nanomaterials cover all shapes as shown in Figure 2. However, only eNanoMapper and NR have all four types of shapes of carbon-based nanomaterials: nanoparticles, nanotubes, nanofilms, and nanocomposites. NKB and NBIK contain only carbon-based nanoparticles. NIL and caNanoLab include nanoparticles, nanotubes, and nanofilms, but not nanocomposites of carbon-based nanomaterials.

**Figure 3:**
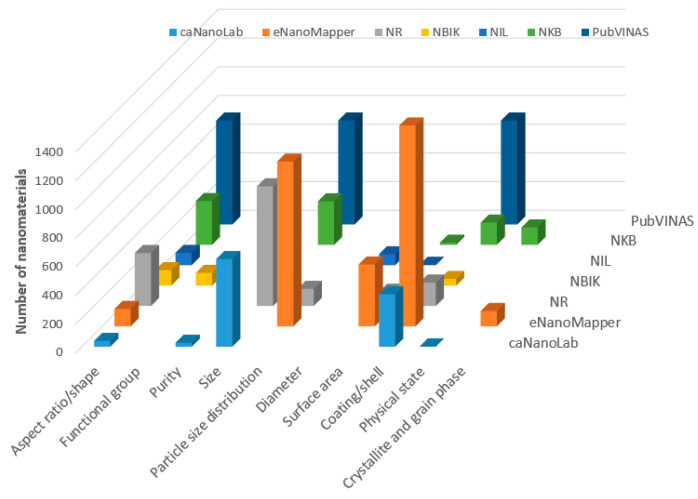
Number of nanomaterials (*z*-axis) with structure characterizations (indicated on the *x*-axis) in the seven databases (depicted in different colors and marked on the *y*-axis).

The quantities of nanomaterials for each type of structural characterization in the seven databases are summarized in Figure 3. Aspect ratio/shape information is provided in all the seven databases. Except NIL, the other six databases cover coating/shell information and size-related information, such as size or size distribution. However, NIL has diameter information, which is not included in other databases. Surface area information is stated in the databases of eNanoMapper, NR, NKB and NIL. The purity property is only mentioned in caNanoLab and NR. It is noteworthy that caNanoLab and NKB also have physical state information. For functional group information, it is provided only by NBIK, and crystallite and grain phase information is included only in the eNanoMapper database.

**Figure 4:**
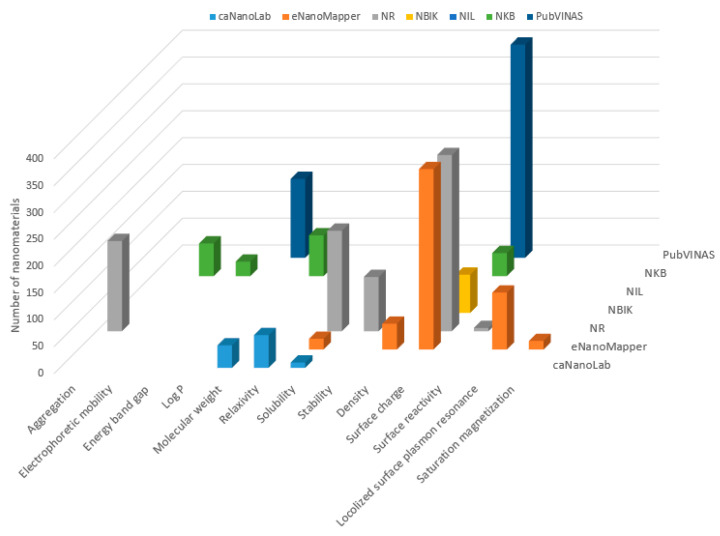
Number of nanomaterials (*z*-axis) with physicochemical properties (indicated on the *x*-axis) in the seven databases (depicted in different colors and marked on the *y*-axis).

The quantities of nanomaterials for each type of physicochemical property in the seven databases are summarized in Figure 4. It is worth mentioning that physicochemical properties are sparsely scattered in the seven databases. Most of the physicochemical properties are included in only one database: electrophoretic mobility and energy band gap in NKB; aggregation, stability, and surface reactivity in NR; density, localized surface plasmon resonance, and saturation magnetization in eNanoMapper; Log P in PubVINAS; and relaxivity in caNanoLab. Molecular weights are provided in both caNanoLab and NKB for a small number of nanomaterials. Solubility data are included in caNanoLab, eNanoMapper, and NR for a small portion of the nanomaterials. Surface charge is the physicochemical property that is included for most the nanomaterials in five of the seven databases (eNanoMapper, NR, NBIK, NKB, and PubVINAS). It is noteworthy that caNanoLab also has surface charge. However, users need to zoom in on the record for each nanomaterial to find surface charge. It is hard to find the number of nanomaterials that have surface charges. Therefore, caNanoLab was not included in the surface charge discussion as shown in Figure 4.

**Figure 5:**
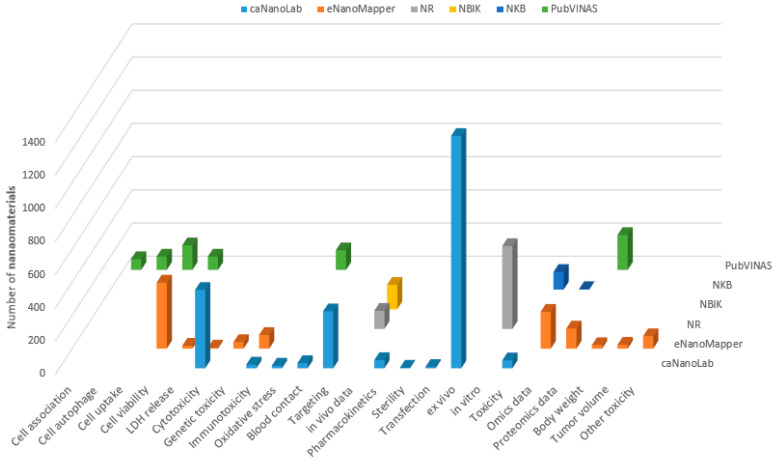
Number of nanomaterials (*z*-axis) with biological activity data (indicated on the *x*-axis) in the six databases (depicted in different colors and marked on the *y*-axis).

The biological properties were also compared among the seven databases. It is noted that caNanoLab and eNanoMapper databases have more biological data points than the other databases, while NIL does not have biological data in a searchable field. Thus, it is not included in the subsequent comparison. The quantities of nanomaterials for 23 common types of biological characterizations in six databases are shown in Figure 5. Similar to the physicochemical properties, the biological data are very sparse in the six databases. It is noticeable that caNanoLab and eNanomapper not only contain more nanomaterials than the other databases (Table 2), but also include more biological data than the other databases. Interestingly, PubVINAS also has rich information of biological characterizations. The biological data contained in the other three databases (NR, NBIK, NKB) are in small amounts with very few types.

The five most basic database functionalities (browse, search, filter, data export, and data upload) for the seven nanomaterial databases are summarized in Table 3. All seven databases provide browse function for users to examine the database content. Except for PubVINAS without searching and NIL not having filtering, the databases have diverse searching and filtering functions for users to narrow down specific nanomaterials, structure characterizations, physicochemical properties, and biological data of interest. PubVINAS, caNanoLab, eNanoMapper, and NKB support data import and export so users can upload nanomaterials and related data or export nanomaterials and associated data of interest, which facilitates data sharing within the nanoscience community.

## 4. Perspectives

According to our analysis, the NR, eNanoMapper, and PubVINAS databases contain more nanomaterials, as well as structure characterizations and physical chemical properties, and are useful in designing new nanomaterials and studying physicochemical properties of nanomaterials. Regarding biological experiments and relevant protocols, caNanoLab, eNanoMapper, and PubVINAS include more data, which provide rich information for risk assessment of nanomaterials and safety evaluation of nanomaterial-containing products.

Undoubtedly, increasing data abundancy in the nano-field has driven the effort in database development within the scientific community. Publicly accessible databases are key resources for learning and retrieving field-specific knowledge. Additionally, databases may promote the development of modern computational nanotechnology, such as nano-informatics modeling studies that target rational nanomaterial design. However, the sizes of current nanomaterial databases are relatively small compared to the abundance of data generated in the nanoscience field, with only a few thousand entries at best as we can see from Table 1. This phenomenon reflects the inefficiency of data sharing after data generation in different laboratories, showing more efforts are required in the assistance of data collection and deposition into public databases. Therefore, more user-friendly tools should be provided by each database to promote data sharing. In addition, literature data mining would also be a good way to collect and analyze nano-related data. For example, a meta-analysis approach that employed decision trees with feature selection algorithms was developed to assemble and generalize the published nanoparticle cytotoxicity data [55]. Similar works by combining data mining and machine learning algorithms to predict the cytotoxicity of nanoparticles were also published [56,57,58,59,60]. Disadvantages to data mining also exist. For example, there is strong dependence on the historical data and the quality of the knowledge obtained through data mining. Thus, solving issues like the inconsistencies coming from different data resources would be of great significance.

Unlike PubChem (pubchem.ncbi.nlm.nih.gov, accessed on 16 February 2021) and PDB (www.rcsb.org, accessed on 16 February 2021), which are two big, well-structured databases in the fields of chemistry and biology, to date there is no comparable nanomaterial database. In the PubChem database, information such as physicochemical properties, structural annotation, and available bioactivities of chemicals are provided [61]. PDB provides 3D structures for a large number of biological macromolecules [62]. To fill gaps in the nanomaterial databases, one of the necessary steps is to provide nanostructure annotation. Furthermore, data completeness and compliance should be evaluated by setting the proper annotation and deposition standards. In short, large nanomaterial databases such as PubChem and PDB or specific databases such as EADB [63] are needed to facilitate nanoscience research.

Data quality is the heart of science, and many efforts have been made to ensure and improve data quality in other fields such as genetics [64,65], genomics [66,67,68], and food science [69]. According to the recently established FAIR (finable, accessible, interoperable, and reusable) guiding principles, the reuse of nanosafety data involves data quality issues such as different levels of processed data, poorly described (meta)data, and limited harmonized reporting formats and tools for data integration and interpretation [70]. Notably, these issues are also the technical challenges that scientists face when building a nanomaterial database. The lack of consistent identification of nanomaterials, the variations in the levels of data processing, and different output formats are common issues when working with multiple databases. Therefore, nanomaterial database developers should make certain rules for determining the accuracy and validity of data to guarantee data quality. For example, using a standard framework or assay for nanotoxicity evaluation could minimize the generation of conflicting and debatable results and harmonize the reporting endpoints, which would make the data interpretation and integration more convenient and efficient. Moreover, relevant nanotoxicity data are still limited, especially the health effects of nanomaterials with low doses, long exposure times, and complex matrix components, which should be emphasized [71].

While rapid developments in the nano-field have brought hope for a potential industrial revolution [72], they have also raised serious concerns regarding their safety, ethics, and regulation [73,74]. Consequently, consistent and concerted efforts from researchers, database stewards, and publishers to promote the development of nanotechnology are still required.

## Figures and Tables

**Table 1:** Popular databases of nanomaterials (all the web links were accessed on 16 February 2021).

Database	Website	Records	Remark
caNanoLab	https://cananolab.nci.nih.gov/	1383	Nanotechnology in biomedicine
eNanoMapper	https://data.enanomapper.net/	2380	Safety assessment of nanomaterials
NR	https://nanomaterialregistry.net/	2031	Physicochemical properties
Nanowerk	https://www.nanowerk.com/	3785	Commercially available nanomaterials
NBIK	http://nbi.oregonstate.edu/	147	Exposure effect in embryo zebrafish
NIL	http://nanoparticlelibrary.net/	88	Physicochemical characteristics
NKB	https://ssl.biomax.de/nanocommons/	598	Nano-safety knowledge infrastructure
PubVINAS	http://www.pubvinas.com/	725	An online nano-modeling tool

**Table 2:** Nanomaterials in the seven databases.

	Carbon	Lipid	Metal	Metal Oxide	Polymer	Semiconductor	Other
caNanoLab	78	97	143	272	528	73	192
eNanoMapper	120	42	723	150	513	226	606
NR	210	2	551	612	190	235	231
NBIK	4	0	47	22	33	34	7
NIL	17	0	15	13	0	25	18
NKB	31	0	164	96	0	50	257
PubVINAS	147	0	456	32	56	34	0

**Table 3:** Nanomaterials in the seven databases.

Function	caNanoLab	eNanoMapper	NR	NBIK	NIL	NKB	PubVINAS
Browse	Yes	Yes	Yes	Yes	Yes	Yes	Yes
Search	Yes	Yes	Yes	Yes	Yes	Yes	
Filter	Yes	Yes	Yes	Yes		Yes	Yes
Export	Yes	Yes	Yes	Yes		Yes	Yes
Upload	Yes	Yes				Yes	Yes
